# The Predictive Model of Oral Squamous Cell Survival Carcinoma: A Methodology of Validation

**DOI:** 10.1155/2021/5436894

**Published:** 2021-11-25

**Authors:** Wan Muhamad Amir W Ahmad, Muhammad Azeem Yaqoob, Nor Farid Mohd Noor, Farah Muna Mohamad Ghazali, Nuzlinda Abdul Rahman, Liszen Tang, Nor Azlida Aleng, Mohammad Khursheed Alam

**Affiliations:** ^1^School of Dental Sciences, Health Campus, Universiti Sains Malaysia (USM), 16150 Kubang Kerian, Kota Bharu, Kelantan, Malaysia; ^2^Faculty of Medicine, Universiti Sultan Zainal Abidin (UniSZA), Medical Campus, Jalan Sultan Mahmud, 20400 Kuala Terengganu, Terengganu, Malaysia; ^3^School of Mathematical Sciences, Universiti Sains Malaysia (USM), 11800 Pulau Pinang, Malaysia; ^4^Faculty of Ocean Engineering Technology and Informatics, Universiti Malaysia Terengganu (UMT), 21030 Kuala Nerus, Terengganu, Malaysia; ^5^College of Dentistry, Jouf University, Sakaka, Saudi Arabia; ^6^Department of Dental Research Cell, Saveetha Dental College and Hospitals, Saveetha Institute of Medical and Technical Sciences, Chennai, India

## Abstract

**Background:**

Cancer is primarily caused by smoking, alcohol, betel quit, a series of genetic alterations, and epigenetic abnormalities in signaling pathways, which result in a variety of phenotypes that favor the development of OSCC. Oral squamous cell carcinoma (OSCC) is the most common type of oral cancer, accounting for 80–90% of all oral malignant neoplasms. Oral cancer is relatively common, and it is frequently curable when detected and treated early enough. The tumor-node-metastasis (TNM) staging system is used to determine patient prognosis; however, geographical inaccuracies frequently occur, affecting management.

**Objective:**

To determine the additional relationship between factors discovered by searching for sociodemographic and metastasis factors, as well as treatment outcomes, which could help improve the prediction of the survival rate in cancer patients. *Material and Methods*. A total of 56 patients were recruited from the ambulatory clinic at the Hospital Universiti Sains Malaysia (USM). In this retrospective study, advanced computational statistical modeling techniques were used to evaluate data descriptions of several variables such as treatment, age, and distant metastasis. The R-Studio software and syntax were used to implement and test the hazard ratio. The statistics for each sample were calculated using a combination model that included methods such as bootstrap and multiple linear regression (MLR).

**Results:**

The statistical strategy showed R demonstrates that regression modeling outperforms an R-squared. It demonstrated that when data is partitioned into a training and testing dataset, the hybrid model technique performs better at predicting the outcome. The variable validation was determined using the well-established bootstrap-integrated MLR technique. In this case, three variables are considered: age, treatment, and distant metastases. It is important to note that three things affect the hazard ratio: age (*β*_1_: -0.006423; *p* < 2*e* − 16), treatment (*β*_2_: -0.355389; *p* < 2*e* − 16), and distant metastasis (*β*_3_: -0.355389; *p* < 2*e* − 16). There is a 0.003469102 MSE for the linear model in this scenario.

**Conclusion:**

In this study, a hybrid approach combining bootstrapping and multiple linear regression will be developed and extensively tested. The R syntax for this methodology was designed to ensure that the researcher completely understood the illustration. In this case, a hybrid model demonstrates how this critical conclusion enables us to better understand the utility and relative contribution of the hybrid method to the outcome. The statistical technique used in this study, R, demonstrates that regression modeling outperforms R-squared values of 0.9014 and 0.00882 for the predicted mean squared error, respectively. The conclusion of the study establishes the superiority of the hybrid model technique used in the study.

## 1. Introduction

Oral squamous cell carcinomas (OSCC) are the sixth most common malignant tumor [1], and they are a fatal oral cavity disease that accounts for up to 50% of all deaths and multiple factors playing a role in survival rate such as T4 stage diagnosis and late age [2]. These cancers account for approximately 2 to 5% of all cancer cases worldwide, with Asia having the highest prevalence [3, 4]. Despite recent advances in therapeutic strategies, the overall survival rate has remained constant over the last few decades [2]. Smoking, tobacco use, alcohol consumption, paan, betel quid, viral stimuli, and some genetic and epigenetic changes are all factors in the development of oral cancers [5–7]. In GLOBOCAN 2020 that estimated the oral cancer incidence, an estimated 377,713 new cases of oral cavity cancer were reported worldwide, along with an increase in mortality, with 177,757 deaths from the disease [8]. According to the World Health Organization (WHO), the incidence of oral cancer is 3.0 per 100,000 when age-standardized to the Malaysian population [9].

Oral cancer is most common in Indian females, with an ASR of 10.2/100,000 female populations. According to data published in April 2011 by the Oral Cancer Research and Coordinating Center (OCRCC) Malaysia, oral cancer deaths in Malaysia totaled 1587, accounting for 1.55 percent of all deaths. Malaysia ranks 14th in the world with an age-adjusted death rate of 7.72 per 100,000 people in 2017 (OCRCC). A previous study in Kelantan found prognostic factors for mortality of oral cancer patients. Being elderly and male and having history of alcohol consumption and an advanced stage of cancer at the time of diagnosis, as well as not receiving treatment, all contributed to a poor prognosis [10]. The use of betel quid chewing, tobacco, and alcohol consumption have all increased the risk of oral cancer in many developing countries [6]. A major causative role for the human papillomavirus (HPV) in OSCC has been established in several recent studies [11–13]. In clinical settings around the world, the human papillomavirus (HPV) is a major source of concern and public burden. Tonsils and the base of the tongue are two of the most common sites for HPV-related cancers [14].

The presence of HPV in oropharyngeal cancers has been documented in multiple studies. Oral cancer is more common than oropharyngeal cancer, but HPV is less common in the mouth. According to a previous assessment of HPV 16 carcinogenicity by the International Agency for Research on Cancer (IARC), there is a hierarchy of evidence for HPV 16 carcinogenicity in the oral cavity and oropharynx, but only a small amount of evidence for laryngeal cancer. According to a recent WHO report on the classification of head and neck tumors with HPV carcinogenesis, 3 percent of OSCC cases are linked to HPV infection [15]. Ndiaye et al., for example, looked into the prevalence of HPV and discovered that HPV DNA prevalence estimates for oral cancers were 24.2 percent [16]. The role of HPV in the oral cavity has already been established, but the prevalence of HPV varies significantly depending on anatomical location, ethnicity, detection methods, and geographic location. There are only a few studies in the literature that have determined the prevalence of HPV in OSCC in the Malaysian population [12, 17].

The tumor-node-metastasis (TNM) staging system is currently one of the most effective prognostic tools for tumor survival [12]. Furthermore, patients' sociodemographic and clinical characteristics, such as age, gender, and smoking habits, are taken into account when determining the best therapeutic strategy, as well as the risk of complications and prognostic value of a variety of cancers [7]. These various identification factors are linked to a poor prognosis, posing a serious problem in the treatment of OSCC. Many different clinicopathological parameters have been studied as independent prognostic factors in patients with OSCC in previous studies, including age, smoking history, TNM staging tumor spread in cervical lymph nodes, tumor size, and microvascular invasion [2]. Nonetheless, previous studies have only looked at a small number of risk factors that could affect prognosis, possibly due to a lack of data on OSCC prognosis in the Kelantan population. As a result, we looked into the possible relationship between sociodemographic and metastasis factors and treatment outcomes in this study.

## 2. Materials and Methods

### 2.1. Data Collection

This research evaluated data from a sample of patients visiting the ambulatory clinic at the Hospital Universiti Sains Malaysia (USM). A total of 56 individuals were recruited in the trial.  Table 1 summarizes the data description of the chosen variables for the research.

### 2.2. Study Design

This is a combination of a retrospective study with advanced computational statistical modeling techniques, which more focus on the methodology development of the multiple linear regression. The study case was illustrated by hazard ratio (*Y*), treatment (*X*_1_), distant metastasis (*X*_2_), and age (*X*_3_). This developed methodology was based on the testing and training dataset, MSE-predicted, and the accuracy value of the predicted analysis. The Universiti Sains Malaysia Research Ethics and Committee (Human) (USM/JEPeM/16050184) approved the study. The patient's privacy and medical condition are both protected.

### 2.3. Modeling of Computational Biometry

The data were evaluated for hazard ratio. The data were examined using the R-Studio software and the syntax that was implemented. Using this approach, the advanced strategy is a combination model that incorporates methods such as bootstrap and multiple linear regression (MLR). Using this technique, the data is divided into two distinct groups, which are referred to as the training data and the testing data, respectively. For the modeling purpose, the training data will be utilized, and for the validation reason, the testing data will be used.

### 2.4. Bootstrap

**Figure 1:**
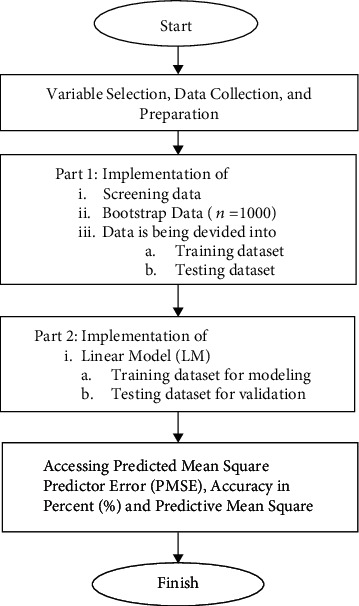
Flowchart of the proposed statistical modeling.

Bootstrap starts by choosing a sample of the population at random and then calculating sample statistics for that sample. Following many replications of the original samples, the bootstrap creates a pseudopopulation via the use of several substitution samples, which are then replicated several times more. Following many replications of the original samples, the bootstrap creates a pseudopopulation via the use of several substitution samples, which are then replicated several times more. Random sampling generates samples that are not similar to the original sample when replacement is used. The bootstrap calculates statistics for each sample as it is drawn with replacement, and it is used to draw samples with replacement [18, 19]. The result for the model is displayed in Table 2. The linear regression is fitting through the R software. The full step by step method is given in Figure 1.

The complete process for creating the statistical model is shown in Figure 1. Before starting the data collecting procedure, the clinical expert determines which variables will be utilized. The study's merit is that it examines a model that considers clinically relevant factors. Following the preparation of the data, the development of a bootstrapping technique will take place. The bootstrap method generates a sample of the same size as the original sample, but with each observation repeated several times and others discarded [18, 19].

## 3. Results

This current study is aimed at investigating the performance of the MLR using the recently created approach, which takes into account both the training and testing datasets. The optimal model for MLR was determined by the combination of chosen variables that generates the lowest predicted MSE as determined by the MLR algorithm.

### 3.1. Regression Modeling

Results of multiple linear regression using a training dataset are shown in Table 2.

In this section, the variable validation was determined utilizing the established bootstrap-integrated MLR technique. There are three selected variables in this case, which are age, treatment, and distant metastasis. The hazard ratio has been significantly influenced by three factors: age (*β*_1_: -0.006423; *p* < 2*e* − 16), treatment (*β*_2_: -0.355389; *p* < 2*e* − 16), and distant metastasis (*β*_3_: -0.355389; *p* < 2*e* − 16). MSE for the linear model has a value of 0.003469102 in this case. The outcome in this study is a hazard ratio value, which is a dependent variable. The train to test split is 70 : 30, which means that 70% of the data is accessible for modeling, and 30% is available for testing. Results of the multiple regression modeling are summarized in Table 2. The model is shown below.

(1) $$\begin{array}{c} \text{Hazard ratio}=0.702051+-0.006423\text{ age}+-0.355389\text{treat}+0.235307\text{dist}. \end{array}$$

### 3.2. Model Evaluation of the Model

In this scenario, the model assessment can be derived from the forecast value. The prediction's quality will be determined by comparing the actual and predicted values. The test dataset will be used to evaluate the model that was obtained from the training data set. When comparing the actual and predicted data, the distance prediction will be used. Through the use of the model assessment approach, which is provided in the form of the R syntax, it will be possible to determine whether or not the produced method was successful.  Table 3 shows the result for the “actual” and “predicted” values using the proposed methodology.

There is not much of a difference between the “actual” and “predicted” values. The results of a paired sample *t*-test showed no statistically significant differences.

Table 4 shows the results of the suggested model's “actual” and “predicted” values. There was no statistically significant difference between “actual” and “predicted,” according to the findings. This indicates that the proposed model is the best.

## 4. Discussion

We were able to obtain and harmonize the hybrid method, resulting in a highly accurate and reliable model. We successfully used the proposed method, and this is very useful to estimate the probabilities of events (predict the odds of being a case). This model is an exponential equation-based model. Previous studies show nonlinear prognostic survival cancer models. Nonlinear models are sometimes consistent and sometimes not. Nonlinear regression models have limitation such as in estimation, calculation procedures are more complicated and less accurate and less precise for each predictor variable and outcome. Hazard ratios are commonly used in prospective studies as a measure of the strength of an association. It is the outcome of comparing the hazard function among those who have been exposed to the hazard function and those who have not. A hazard ratio of 1 indicates no association, while a hazard ratio greater than 1 indicates an increased risk, and a hazard ratio below 1 indicates low risk. The Cox regression coefficient is used to calculate the hazard ratios. The hazard ratio can be thought of as an estimate of relative risk, which is the likelihood of an event (or the development of a disease) based on exposure. The ratio of the probability of an event occurring in the exposed group versus the control (nonexposed) group is known as relative risk. According to the study's findings, we assessed 56 patients, focusing on their age, treatment received, and distant metastasis. The findings revealed that distant metastasis is the most important factor influencing the patients' hazard ratio. The development of methodologies for MLR is a major focus of this paper. The data was divided into 70% for the training dataset and 30% for the testing dataset. The main objectives were to create, test, and validate a regression modeling methodology. The main goal of this project was to combine the bootstrapping procedure with MLR to develop and implement techniques in the field of medical statistics. The variable selection process incorporates clinical expert opinion. The bootstrap method creates a mega file from the initial data set at the beginning of the operation. On the other hand, the bootstrap procedure generates a large file replacement sample. Thirdly, the bootstrap method generates and saves statistical samples. Fourthly, the bootstrap method repeats this process iteratively, sometimes thousands of times. The fifth stage is used to prepare the data for the subsequent procedure. The R syntax algorithm makes it possible to integrate the application with the methodology concept.

The first step is to consult with a professional when selecting variables. Following that, the bootstrap will be applied to the selected data. Data for training and testing will be kept separate. The R syntax algorithm connects the application to the concept of method-based methodology. The first step is to choose variables with the support and advice of a medical professional. The data will then be subjected to the bootstrap procedure. At this point, 70% of the bootstrap data will be designated as a training dataset, while 30% will be designated as a testing dataset. The training dataset will be used to build the model, while the validation dataset will be used to validate it. The smallest mean square error will be an indicator of a successful model. The following formula was calculated based on the training and test sets. It is preferable to minimize PMSE (which would require obtaining a result with the lowest PMSE value). The results of the study led to the best possible results for the decision-maker. The proposed methodology resulted in extremely successful linear modeling due to the incorporation of statistical formulations, computation using the developed R syntax, and the use of the MLR package. The most difficult tasks are selecting appropriate input parameters, preparing data for linear modeling, and standardizing it.

## 5. Conclusion

In this work, a hybrid approach that incorporates bootstrapping and multiple linear regression will be designed and thoroughly validated. This methodology's R syntax was created to ensure that the researcher fully comprehended the illustration. In this study, hazard ratios are the dependent variable, while age, treatment, and distant metastasis are the independent variables. As a result of the developed model, factors emerged as the most significant factors. The greater the R square (or the greater the value), the more precise the model was created, according to regression theory. Aside from that, the smallest predictive model can be used to determine the goodness of fit of a test. In this case, a hybrid model demonstrates that this important conclusion allows us to better understand the utility of the hybrid method and its relative contribution to the outcome. The statistical strategy proposed in this study in R demonstrates that regression modeling outperforms an R-squared value of 0.9014, and a predicted mean squared error is given as 0.00882. The study's conclusion proves that the hybrid model technique proposed in the study is superior.

## Figures and Tables

**Code 1:**
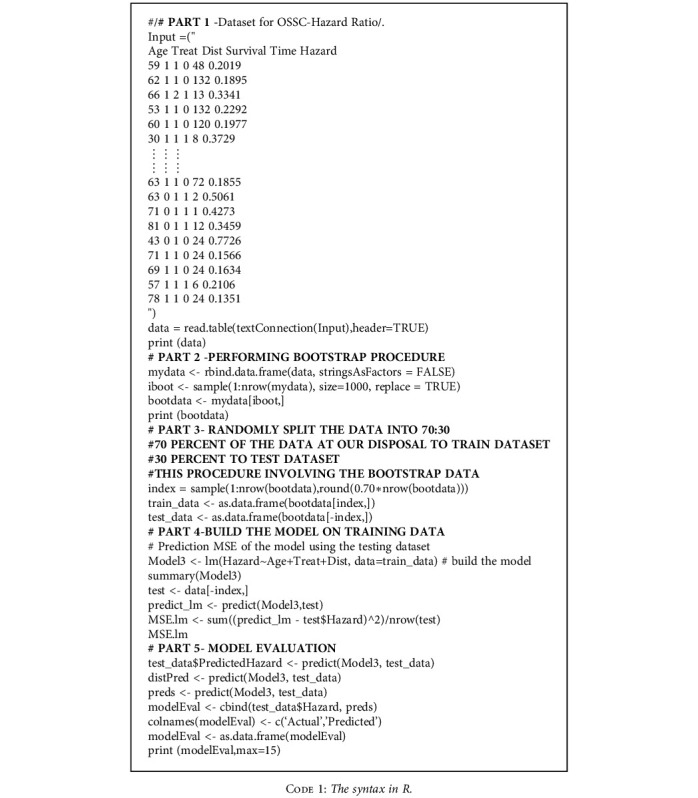
The syntax in R.

**Table 1:** Data description of the selected blood profile.

Variable	Code	Description
HR	*Y*	Hazard ratio
Treatment	*X*_1_	Treatment received 0: no treatment 1: received treatment
Distant metastases	*X*_2_	Distant metastasis is the leading cause of tumor-related death from oral cancer 0: no 1: yes
Age	*X*_3_	Age in years

**Table 2:** Result of multiple linear regression with combining the bootstrap method training and testing dataset.

Variable	Estimate	Std error	*t*-value	*p* value
(Intercept)	0.702051	0.012504	56.15	<2*e* − 16^∗∗∗^
Age	-0.006423	0.000160	-40.15	<2*e* − 16^∗∗∗^
Treatment	-0.355389	0.005228	-67.97	<2*e* − 16^∗∗∗^
Distant metastasis	0.235307	0.005798	40.59	<2*e* − 16^∗∗∗^

Multiple linear regression was applied. Significant at the level of 0.05.

**Table 3:** The “actual” and “predicted”.

Actual	Predicted
0.1855	0.171548
0.2019	0.199609
0.3927	0.458991
0.2761	0.359267
0.1566	0.115426
0.1977	0.192594

**Table 4:** Summary of “actual” and “predicted” value of the proposed model.

Paired samples test	
Variables	Mean (SD)
Actual value of hazard ratio	0.228000 (0.081394)
Predicted value of Hazard ratio	0.238426 (0.123108)
*T* statistics (df)	-0.600377 (6)
*p* value	0.570221
Paired sample correlation (*ρ*)	0.981489
*p* value	*p* < 0.05^∗^

^∗^Significant at the level of 0.05. Paired samples *t*-test was applied. Assumption normality is fulfilled.

## Data Availability

Available within the manuscript.
